# Metamorphic microdiamond formation is controlled by water activity, phase transitions and temperature

**DOI:** 10.1038/s41598-021-87272-1

**Published:** 2021-04-08

**Authors:** J. Kotková, Y. Fedortchouk, R. Wirth, M. J. Whitehouse

**Affiliations:** 1 Czech Geological Survey, Klárov 3, 118 21 Prague 1, Czech Republic; 2 Department of Geological Sciences, Masaryk University, Kotlářská 2, 611 37 Brno, Czech Republic; 3 Department of Earth and Environmental Sciences, Dalhousie University, Halifax, NS B3H 4R2 Canada; 4 Interface Geochemistry, GFZ German Research Centre For Geosciences, Telegrafenberg, C-120, 14473 Potsdam, Germany; 5 Department of Geosciences, Swedish Museum of Natural History, Box 50007, 104 05 Stockholm, Sweden

**Keywords:** Geochemistry, Mineralogy, Petrology

## Abstract

Metamorphic diamonds hosted by major and accessory phases in ultrahigh-pressure (UHP) metamorphic terranes represent important indicators of deep subduction and exhumation of continental crust at convergent plate boundaries. However, their nucleation and growth mechanisms are not well understood due to their small size and diversity. The Bohemian microdiamond samples represent a unique occurrence of monocrystalline octahedral and polycrystalline cubo-octahedral microdiamonds in two different metasedimentary rock types. By combining new and published data on microdiamonds (morphology, resorption, associated phases, carbon isotope composition) with P–T constraints from their host rocks, we demonstrate that the peak P–T conditions for the diamond-bearing UHP rocks cluster along water activity-related phase transitions that determine the microdiamond features. With increasing temperature, the diamond-forming medium changes from aqueous fluid to hydrous melt, and diamond morphology evolves from cubo-octahedral to octahedral. The latter is restricted to the UHP-UHT rocks exceeding 1100 °C, which is above the incongruent melting of phengite, where microdiamonds nucleate along a prograde P–T path in silicate-carbonate hydrous melt. The observed effect of temperature on diamond morphology supports experimental data on diamond growth and can be used for examining growth conditions of cratonic diamonds from kimberlites, which are dominated by octahedra and their resorbed forms.

## Introduction

Most diamonds form at the base of the lithospheric roots of continental cratons where they grow within peridotite and eclogite lithologies during metasomatism by mantle fluids. Microdiamonds of UHP metamorphic terranes are found in metasedimentary rocks with continental crustal affinities that have been subducted to mantle depths in collisional orogens and therefore provide key information about deep subduction processes. These microdiamonds (several tens to, rarely, hundreds of micrometers in size) mostly occur as inclusions within the rock-forming and accessory phases (garnet, clinopyroxene, kyanite and zircon)^1^. Their morphology varies from imperfect skeletal or hopper forms to near-perfect octahedral, cubo-octahedral and cuboid-like crystals^1^ (Table 1); sometimes they develop surface graphitization^2^. The large range of carbon isotope values^3^ of these microdiamonds indicates a variety of carbon sources and, thus, distinct diamond formation mechanisms. The restricted size of these microdiamonds presents challenges to the study and interpretation of their origin. Pioneering investigations^4^ using Transmission Electron Microscopy (TEM) with focused ion beam sample preparation (FIB) and Fourier Transform Infrared Spectroscopy (FTIR) documented both solid and fluid nanoinclusions in microdiamonds whose diverse composition largely reflects that of the host rock and C–O–H fluid^1,5^. Despite numerous studies of UHP microdiamonds from different locations worldwide, the mechanism of their formation and the factors controlling their morphology remain poorly understood^1^.

**Table 1:** Comparison of morphology and mode of occurrence of microdiamonds in metasediments worldwide.

Locality	Dominant morphology	Host rock	Host phase	Mode of occurrence	Major ref
**UHP-LT terranes**					
Lago di Cignana	Cuboid	Oceanic metasediment	Grt	Dia within and adjacent to FI Dia + carbonate + Rt, Dia + H2O-rich FI aqueous liq phase with SO_4_, HCO_3_,CO_3_	^6^
**UHP-HT terranes**					
Tromso Nappe	CUBOID	Garnet ± kyanite-two-mica gneiss	Grt	Single Dia, Dia with carbonate, Gr	^7^
Seve Nappe, Areskutan	Irregular	Kyanite-bearing paragneiss	Grt	Single, with carbonate in Grt CO_2_, CH_4_ often with Gr and carb	^8^
Seve Nappe, Twalaklump	Cuboid	Kyanite-garnet gneiss	Grt	Dia + Qtz, Rt, carbonate, Gr present	^9^
Pohorie	Cuboid	Garnet-kyanite-two-mica gneiss	Grt	Single/in MSI + moissanite, CO_2_, CH_4_	^10^
Central Rhodopes	Cuboid	Garnet-kyanite-two-mica gneiss	Grt	Single Dia, Dia with carb., Gr, rare CO_2_	^11^
**UHP-UHT terranes**					
Kokchetav Massif	Cuboid, cuboctahedron, skeletal	Garnet-biotite gneiss	Grt, Zrn	In MSI with Qtz, Ab, Kfs, Rt, Ap, Tit, micas, Chl, Gr coating	^1, 12^
				Intergranular	^13^
			Zrn	CO_2_ ± H_2_O FI with Dia	^14^
	Octahedral, skeletal	Clinozoisite gneiss	Grt, Zrn ± Ky, Czoi-Qtz sympl	Single, clusters	^15, 16^
Erzgebirge	Cuboid	Garnet-phengite gneiss	Grt, Zrn	in MSI with Phl, Qtz, Pg, Phe, Ap, Rt	^17, 18^
			Zrn	mono/polycrystal. Dia	^4, 5^
			Zrn	polycrystal. Dia, Gr present	^19^
NW Bohemia	Cuboctahedron	Garnet-clinopyroxene UHP rock	Grt, Ky, Zrn	Mono/polycrystal. Dia, + Qtz, Rt, Ap ± carb	^20–22^
	Octahedron	Garnet-kyanite gneiss	Grt, Ky, Zrn	Single	^20^, this work

Cuboid or cubooctahedron term as used by individual authors.*FI* fluid inclusion, *MSI* multiple solid inclusion. Carbonate-bearing rocks from Kokchetav Massif are not included.

A unique occurrence of microdiamonds^20^ with different morphologies and composition of carbon isotopes trapped in similar host minerals from two compositionally different host rock types in the Bohemian Massif within the European Variscan Belt provides an unprecedented opportunity to further constrain the origin of microdiamonds, both locally and worldwide*.* The observation of microdiamonds below the polished surface of the thin section, their octahedral crystal shape, the existence of aggregated crystals, dissolution pits on diamond surfaces, variation of their grain size, and occurrence of coesite, all confirm their in-situ origin, as opposed to any contamination during sample preparation. Here we integrate a study of Bohemian microdiamonds with available data on confirmed microdiamond occurrences in metasedimentary rocks (gneisses) worldwide to discover the origin of UHP diamonds and to constrain the factors controlling their nucleation and growth features. We use diamond crystal morphology, dissolution features, surface graphitization, composition of the material trapped at the diamond-host interface, and the peak P–T conditions of the metasedimentary host rock, to assess the role of different factors that can contribute to diamond nucleation and growth (P, T, water activity, and presence of impurities)^1^. Our results demonstrate that phase transitions involving changes in the water activity during subduction trigger diamond crystallization, and the specific nature of these transitions controls diamond characteristics. Here, we propose a new model for UHP microdiamond crystallization worldwide, making a substantial step forward in the presently accepted concept of microdiamond formation from a supercritical C–O–H fluid/melt^1^.

### Host rocks of the Bohemian microdiamonds

Microdiamond findings in the northwestern part of the Bohemian Massif are restricted to two distinct rock types, which form decimeter to several meters thick layers within diamond-free felsic granulites of leucogranitic composition (leucogranulites^23,24^) both in outcrops and within drill cores^23^. The first diamond-bearing rock, A, is an acidic (68 wt.% SiO_2_), quartzofeldspathic gneiss composed of garnet, kyanite, feldspar, quartz and abundant biotite with a strongly peraluminous composition (A/CNK = molar Al_2_O_3_/CaO + Na_2_O + K_2_O = 1.5) characteristic of former pelitic sediments. The second diamond-bearing rock, B, is an intermediate (57 wt.% SiO_2_) rock consisting of garnet, clinopyroxene, minor kyanite, feldspar, quartz and some biotite^21^, with low A/CNK = 1.0. Variable negative ^176^Hf/^177^Hf ratios of zircon cores suggest a metasedimentary origin of this rock^22^. Diamonds occur in the outer core domain of garnet, kyanite and zircon^22^ in both rock types. The contrasting mineralogy and bulk chemical composition of the two diamond-bearing rocks are accompanied by differences in the diamond features.

From the two host rocks, only rock B contains a variable mineral assemblage and abundant diamond-bearing zircon to allow for evaluation of the P–T evolution. The peak of the UHP-UHT metamorphism was estimated at P–T conditions in excess of 4.5 GPa and 1100 °C^21^. Exhumation along a steep decompressional path at high temperatures (isothermal decompression, ITD) is constrained by conventional thermobarometry, Ti-in-zircon thermometry for diamond-bearing zircon domains, Zr-in-rutile thermometry and thermodynamic modelling^21^ for rock B. Subduction to mantle depth is also confirmed by the similar peak P–T conditions calculated for the associated garnet peridotites^25^.

### Microdiamond features

**Figure 1:**
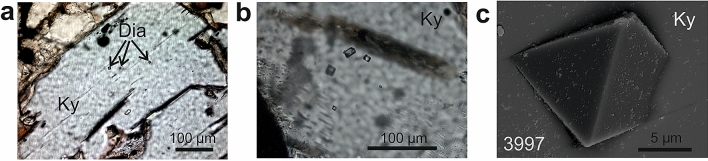
Distribution, mode of occurrence, morphology and surface of microdiamonds, rock A. (**a**,**b**) Photomicrographs showing diamond distribution within kyanite. (**c**) Perfect crystal shape, smooth surface and sharp edges and corners of diamond enclosed in kyanite (SEM). Four-digit number refers to FIB-TEM sample numbers in Supplementary Table S2.

Diamonds in the acidic rock A (Fig. 1a–c) are nearly perfect single octahedra with sharp edges and corners (Fig. 1c, Fig. 3a, Supplementary Fig. S1) and with carbon isotope composition of − 21 to − 22‰ δ^13^C_PDB_ (Supplementary Table S1). They are hosted mostly by kyanite and rare zircon and less frequently by garnet. Microdiamond inclusions in kyanite and zircon are notably smaller (mostly 5–15 µm; Fig. 1a–c, Supplementary Fig. S1) than those in garnet (15–30 µm in size; Fig. 5). The interfaces between diamond and kyanite are sharp, straight and closed, and mostly devoid of any other phases except for a single void (500 × 700 nm; Fig. 3a) where TEM detected amorphous matter containing Ca, Mg, Fe, Zn, Cl and S (Table S1, Fig. 4a, Supplementary Table S2), and an aggregate (2.5 µm × 0.5 µm) with a low-pressure assemblage of Mg-ferrite, quartz and white mica (Fig. 3b). One area of sharp diamond–kyanite interface displays a 200 nm thick layer of disordered graphite likely precipitated from diamond-growth medium during decompression. Some octahedral diamonds hosted by garnet in rock A feature rare negatively oriented triangular etch pits (trigons) that show an association with the outcropping dislocation array evident from TEM images (Fig. 5a,b) and have a steep, pointed-bottom depth profile (Fig. 5c,d).

**Figure 2:**
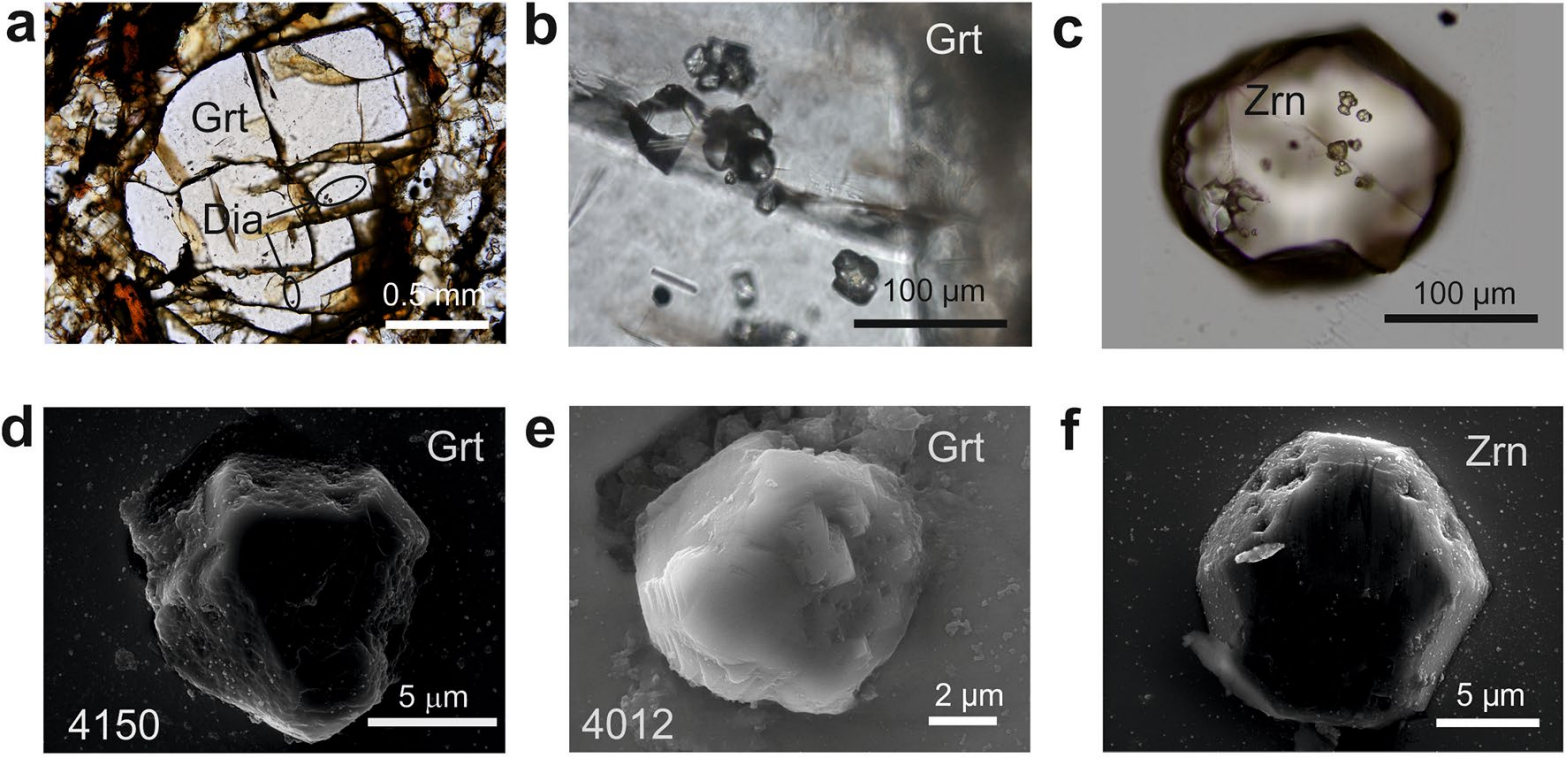
Distribution, mode of occurrence, morphology and surface of microdiamonds, rock B. Photomicrographs showing (**a**) distribution of diamond in the outer core domain of garnet, (**b**) clusters of diamond cubo-octahedra in garnet, and (**c**) distribution of diamond in zircon. (**d**–**f**) SE images showing common irregular shape and rough surface features (**d**,**e**) as well as rare regular (**f**) shape of diamonds. Four-digit numbers refer to FIB-TEM sample numbers in Supplementary Table S2.

**Figure 3:**
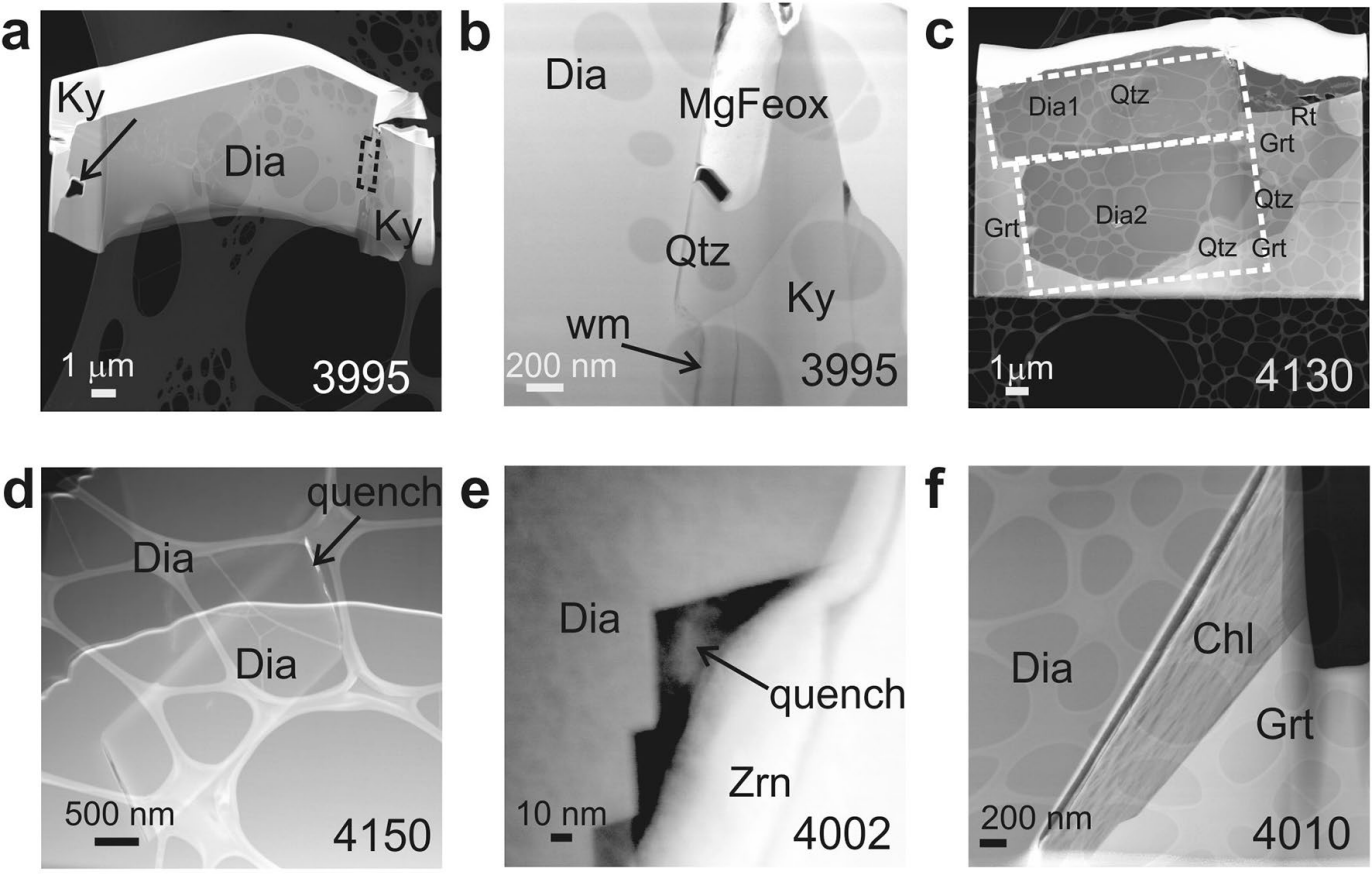
TEM images showing the character of diamond-host interface and internal diamond structure. (**a**,**b**) Rock A. Octahedral diamond in kyanite with sharp, straight boundaries, with a single void (arrow in **a**), and the late assemblage Mg-ferrite-white mica-quartz at diamond-kyanite interface (rectangle in **a**, blowup in **b**: checked by EDX and XRD). (**c**–**f**) Rock B. Polycrystalline internal structure of diamond cuboids in garnet (**c**, two large grains marked by dashed white rectangles) with associated quartz and rutile, and in zircon (**d**). Amorphous quench material in interstitial space (**d**) and in the open zig-zag-shaped interface between diamond and zircon (**e**). (**f**) cuboid diamond hosted by garnet, with open boundary towards chlorite (d_002_-spacing approx. 7 Å) at diamond-host interface. Four-digit numbers refer to FIB-TEM sample numbers in Supplementary Table S2. Note the perforated carbon grid the sample rests on, visible in TEM images.

**Figure 4:**
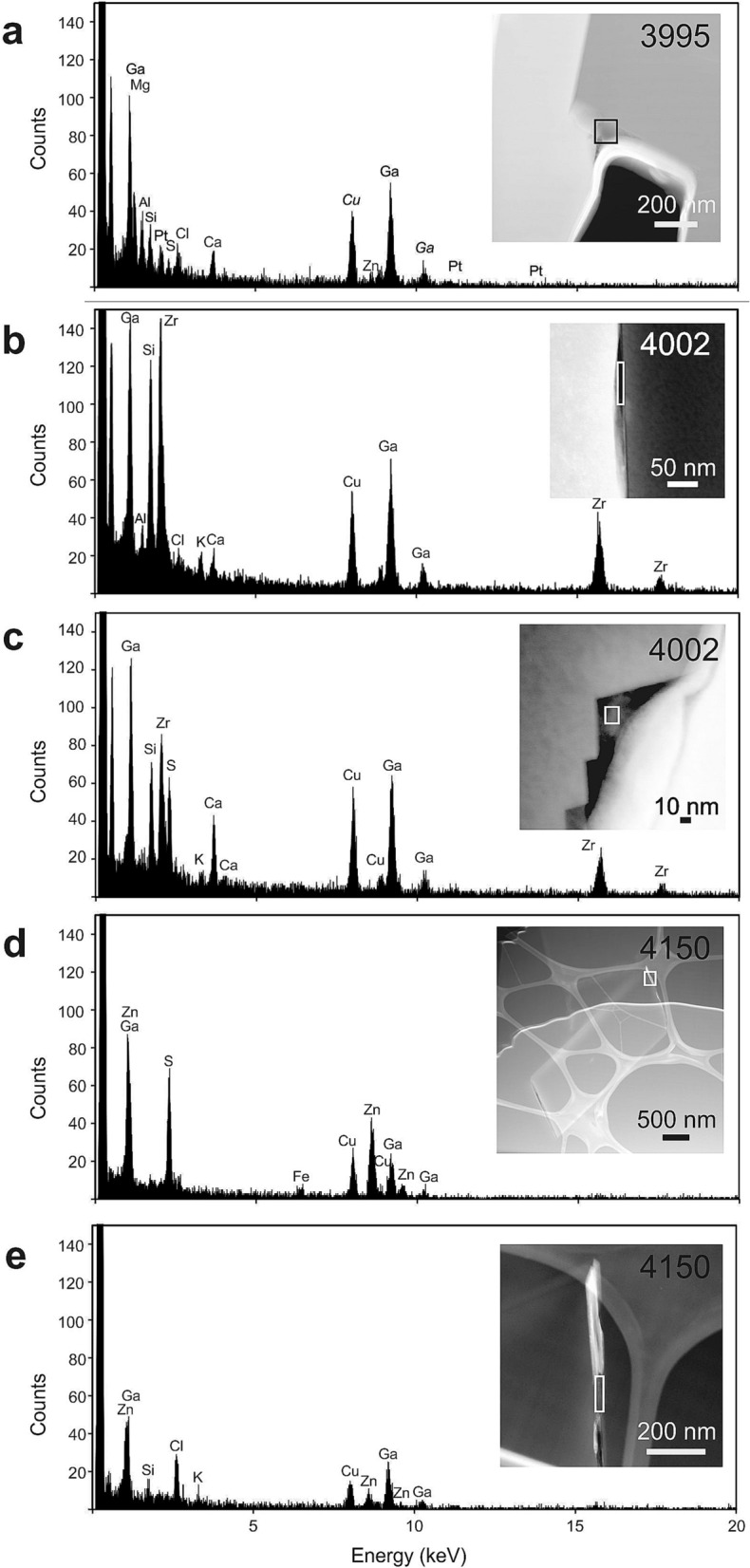
EDX spectra showing composition of the amorphous quench phase (AQP) at diamond-host interface. Ga is from Ga ion implantation during FIB milling, Cu is from the copper support grid and Pt from the strap covering the FIB foil. (**a**) void with relic of AQP at octahedral diamond-kyanite interface (rock A) containing Ca, Mg, Fe, Zn, Cl and S; (**b**) AQP in a gap between diamond and host zircon with Ca, K, Al and Cl; (**c**) relics of AQP in triangular gaps (growth steps on diamond surface) at diamond-zircon interface with Ca, K and S (rock B); (**d**) Zn, Fe and S and (**e**) K, Cl and Zn in intergranular spaces of polycrystalline diamond enclosed in zircon.

Diamonds in the intermediate rock B (5–25 µm in size) have mostly cubo-octahedral to cuboidal shape, with both regular and irregular surface with nanometer-size triangular growth steps (Figs. 2, 3e). They have a light carbon isotope composition (− 26 and − 33‰ δ^13^C_PDB_, Supplementary Table S1). Diamond commonly forms clusters within garnet and zircon (Fig. 2a–c), and individual crystals in rare kyanite. Some diamond grains are polycrystalline (Fig. 3c,d: note the carbon grid in FIB-TEM images). TEM and micro-Raman spectroscopy detected quartz, rutile, apatite and rare CaMg-carbonate along diamond—host mineral interfaces (Fig. 3c). Relics of an amorphous quench phase containing Ca, Al, K, S, Cl, Zn and S have been trapped in nanoscale voids in triangular gaps between the growth steps on diamond surface at the diamond-zircon interface, and in interstitial spaces of polycrystalline diamond aggregates (Figs. 3d,e, 4b–e, Supplementary Table S2). Graphite and chlorite (Fig. 3f) detected at the diamond-host interface continue into the fractures in the host mineral, which implies their secondary origin.

Overall, the phases and composition of the residual material along the interface of both octahedral and cubo-octahedral diamonds in both rock types, containing Ca, Fe, Mg, Na, K, halides and sulfates, are remarkably similar. Notable is that unlike microdiamonds elsewhere^1, 5^, diamonds from both rock types in this study lack any nanometer-sized solid or fluid inclusions in their interior.

### Diamond-forming media: constraints from microdiamonds and whole rocks

The standing paradigm of microdiamond formation from high-density supercritical fluids/melts still does not include a clear link between the character of diamond-forming media and the variability of microdiamond forms, associated phases, and inclusions, observed in UHP metamorphic terranes (Table 1). The documented UHP occurrences show peak conditions over a large T range (600–> 1100 °C; Table 2), including low-T rocks (Lago Cignana), high-T gneisses (Scandinavian Caledonides and Pohorie), and ultrahigh -T rocks (Kokchetav, Erzgebirge, Bohemia). Cubo-octahedra or cuboids are the dominant diamond forms for the whole T range of UHP rocks (Table 1). By contrast, the occurrence of octahedral diamonds is restricted to the Bohemian UHT rocks (rock A from this study) and an unusual UHT zoisite gneiss from Barchi Kol in Kokchetav Massif (Table 1). Cuboidal diamonds occur inside aqueous fluid inclusions along with carbonates^6^ in the low-T rocks (~ 600 °C), and in association with both fluid (CO_2_, CH_4_) and solid phases (carbonates, moissanite)^7,10,11^ in high-T gneisses (750–850 °C). In ultrahigh-T rocks (˃ 1000 °C), diamonds commonly occur within multiphase solid inclusions representing crystallized felsic melt (MSI), in rare cases in the form of intergranular diamonds^13^, and they are associated with CO_2_-bearing fluid inclusions^14^.

**Table 2:** Summary of peak P–T estimates and retrograde paths for diamond-bearing metasediments worldwide.

	Rock type	Peak P (GPa)	Peak T (°C)	Exhumation P–T path	References
**UHP-LT terranes**					
Lago di Cignana	Metasediments	2.7–2.9	600–630	ITD	^26^
	Eclogite*	> 3.2	590–605	ITD	^27^
**UHP-HT terranes**					
Tromso Nappe	Garnet ± kyanite-two-mica gneiss	3.5 ± 0.5	770 ± 50		^7^
Areskutan, Seve Nappe	Kyanite-bearing paragneiss	4.1–4.2	830–840	ITD	^8^
Pohorie**	Garnet-kyanite-two-mica gneiss	3.4 ± 0.25	800 ± 50	near-ITD	^10^
Central Rhodopes	Garnet-kyanite-biotite gneiss	3.5–4.6	700–800	ITD	^11^
**UHP-UHT terranes**					
Kokchetav Massif	Garnet-biotite gneiss	4.5–5	910–1040	near-ITD	^28^
Erzgebirge	Garnet-phengite gneiss	3–5	1000–1100		^29^
NW Bohemia	Garnet-clinopyroxene rock	> 4.5	> 1100	ITD	^21^

*ITD* isothermal decompression. *Peak P–T estimate used in Fig. 4 coming from the associated diamond-free rocks. ** Peak P–T conditions within the range comparable to those of the associated eclogites.

The mode of microdiamond occurrence and P–T constraints from their metasedimentary host rocks provide crucial information to discriminate between fluid and melt diamond growth media. Microdiamonds in the Bohemian UHP-UHT (˃ 1100 °C) rocks in this study occur inside the host minerals with only a minuscule amount of other mineral phases detected along the diamond-host interface. This excludes diamond nucleation and growth within melt or fluid inclusions and suggests their entrapment as syngenetic or protogenetic inclusions by the host mineral phases. Cleavage in the kyanite host next to an undeformed diamond inclusion provides further evidence for diamond entrapment as a crystallized phase.

The residual material trapped along the diamond-host interface containing Ca, Fe, Mg, Na, K, halides, and sulphates in both rock types indicates similar silicate-carbonate fluid/melt growth media for both diamond morphologies in both rock types. Therefore, the effect of the growth medium composition on diamond morphology reported in experiments^30^ is not observed in our rocks.

**Figure 5:**
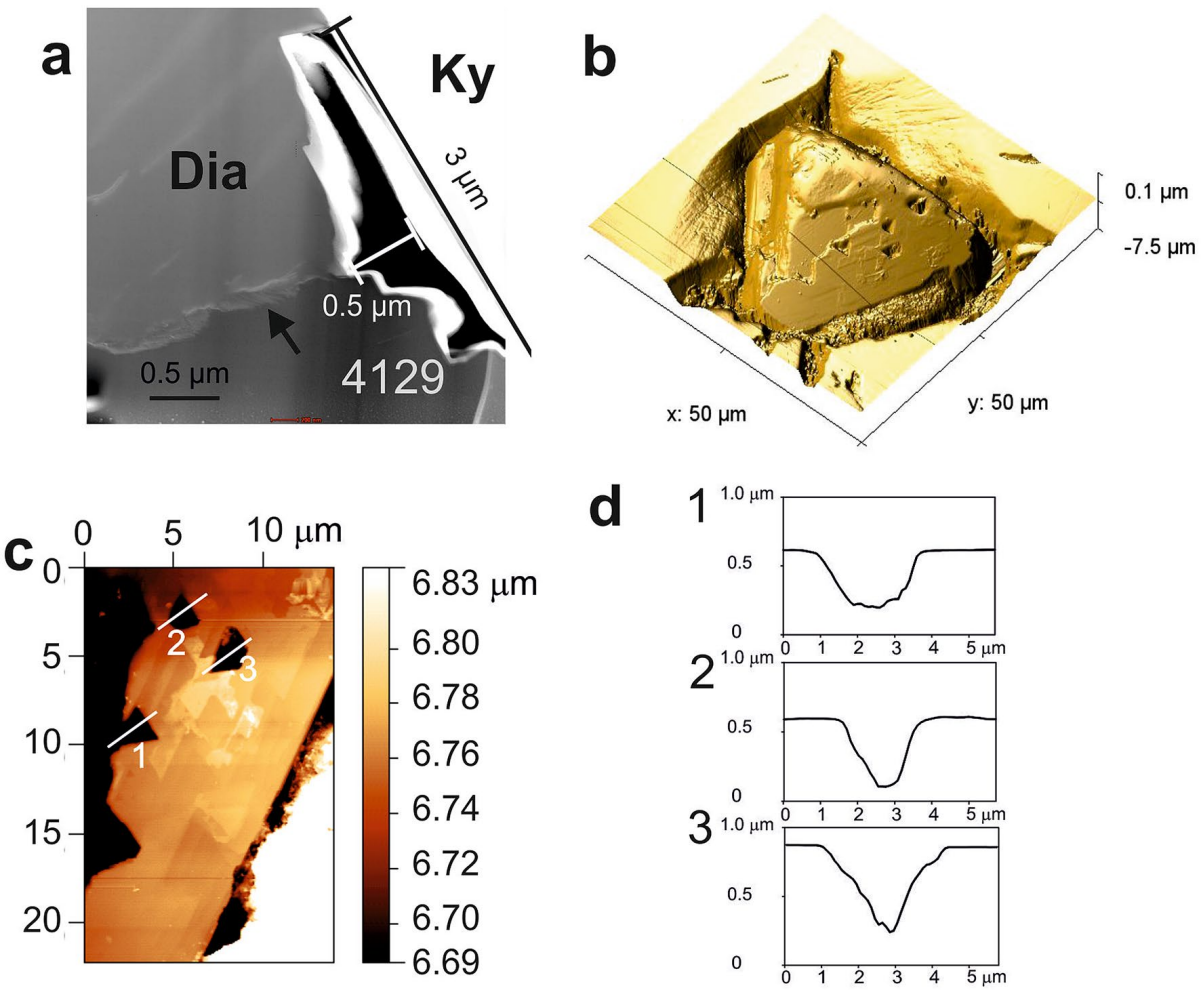
Dissolution phenomena on the surface of octahedral diamond. (**a**) High-angle annular dark-field (HAADF) image of a cross-section through funnel-shape trigonal etch pit on diamond-kyanite interface located at an outcropping dislocation array. (**b**) Negative trigonal etch pits on the octahedral face of diamond (AFM image). (**c**,**d**) Position and depth profiles of the trigons (AFM) which have similar dimensions and shape as the etch pit in (**a**).

Further constraints on the nature of the diamond growth media can be provided by the presence of dissolution trigons on some of the studied diamonds. The dissolution origin of these trigons is confirmed by their association with the outcropping dislocation array on TEM images (Fig. 5a). This dissolution happened after diamond crystallization either before or after its entrapment in the host phases^31^ in the same medium from which diamond grew. Experimental studies^32^ demonstrated a relationship between diamond dissolution morphology and the composition of dissolution medium (solvent), which allows us to examine the growth/dissolution medium of our diamonds. The well-preserved octahedral shape, sharp edges and corners of our diamonds are consistent with dissolution in hydrous silicate-carbonate melt^32^ and contrast with the features characteristic of diamond dissolution in COH fluid: this produces rounded crystal forms^33^ never observed in our samples. The pointed-bottom shape of the trigons on diamonds from rock A (Fig. 5d) indicates that the melt likely had X_CO2_ > 0.5^34^. Experiments show that even at conditions when diamond is very unstable, at 0.1 MPa and at very oxidized conditions with *fO*_*2*_ of 12 log units above FMQ (Fayalite-Magnetite-Quartz) buffer, any diamond etching happens at T > 800 °C^35^. At much higher P, > 4.5 GPa, and fairly reduced conditions in the host rocks here, dissolution would require much higher T similar to the peak conditions estimated for the host rocks in excess of 4.5 GPa and 1100 °C^21^. These are significantly beyond the second critical point (2CP) terminating the wet solidus and above the critical curve for subducted sediments^36,37^, which further supports diamond crystallization from a melt. The estimated peak P–T conditions are also above phengite breakdown that produces hydrous granitic melts with peritectic garnet and kyanite^21^, explaining the uniform Th/U ratios of the diamond-bearing zircon domain^22^.

### Microdiamond source and nucleation: importance of phase transitions related to water activity

Different mechanisms for metamorphic microdiamond formation have been proposed: in-situ formation from internal carbon source, i.e. organic matter^12^, or metasomatism from a mobile C–O–H fluid/melt similar to kimberlitic diamonds^3,38^. The light carbon isotope composition of our diamonds (δ^13^C = − 21 to − 33‰; Supplementary Table S1) and diamond occurrences only within a few distinct lithological layers suggest an internal carbon source from the organic matter (avg. δ^13^C = − 26 ± 7‰)^39^. Buried organic material in sediments is subject to graphitization at increasing P and T conditions^40^. Metamorphic graphite inherits the carbon isotopic composition from its organic precursor^40^ that can also explain the different carbon isotope composition of diamonds in the two rocks as a feature inherited from the source.

Solid-state transformation of graphite into diamond requires high activation energy and significant overstepping of the diamond-graphite transition curve or the presence of catalysts in amounts not available in natural systems^41^. However, in the presence of a fluid or melt, such conversion can happen at pressures close to the diamond-graphite transition curve^42^ through simultaneous graphite dissolution and diamond precipitation driven by the difference in graphite and diamond solubility. Such a process of graphite to diamond transformation requires a constant source of excess carbon and a presence of melt or fluid. The melt produced through phengite breakdown^21,37^ can serve as one such diamond nucleation medium, while the source of excess carbon is maintained when diamond grows in the matrix of a graphite-bearing rock and is enclosed in the host phase after its crystallization from the melt. This indicates diamond crystallization on the prograde path in the studied rocks from the Bohemian Massif. A number of phase changes along the prograde metamorphic path provide mechanisms for spontaneous diamond crystallization: (1) transition from aqueous fluid with higher carbon solubility to hydrous melt with lower carbon solubility over the “wet” solidus below 2CP^37,43^; (2) dehydration melting upon decomposition of phengite at T > 1000 °C^36,37^ which produces a medium for graphite—diamond transformation; (3) crossing the miscibility gap between carbonate and silicate melts^44,45^ which have different carbon solubility. Since the peak conditions estimated for the rocks from the Bohemian Massif ^21^ are significantly above 2 CP and the wet solidus for pelites, but close to the phengite decomposition and the miscibility gap for carbonatitic and silicate melt^37,44,45^, mechanisms 2 and 3 are better applicable to Bohemian rocks, where diamonds form during P–T increase. By contrast, on the retrograde path, decrease of P and T supresses carbon solubility, allowing attainment of saturation and triggering crystallization of diamond^42^. This mechanism can explain microdiamond occurrence inside fluid or melt inclusions in most UHP metamorphic terranes.

### Implications for the microdiamond formation worldwide and ultradeep subduction

**Figure 6:**
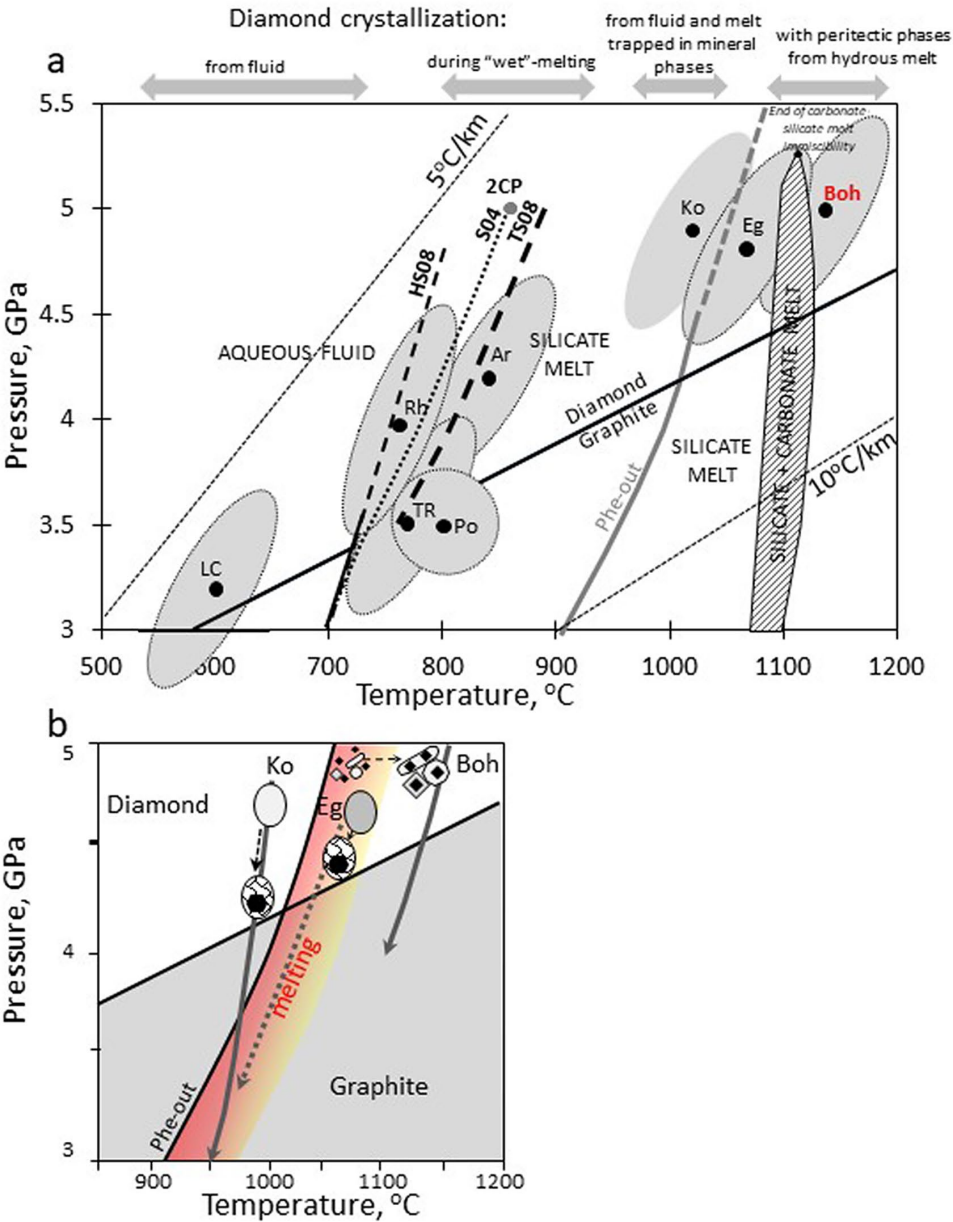
Mechanism of diamond crystallization in UHP terranes. (**a**) P–T peak conditions of diamond-bearing UHP terranes (see Table 2) relative to diamond-graphite transition^47^ and melting reactions for crustal rocks. “Wet” solidus and second critical point (2CP) of pelites: solid line HS08^37^, stippled line S04^43^. Dashed line TS08^45^ is wet solidus of carbonated pelites. Phe-out^37^ marks phengite breakdown. Dashed field: miscibility gap between silicate and carbonate melts^44^. UHP rocks: low-T Lago di Cignana (LC) rocks; intermediate-T gneisses Tromso Nappe (TR) and Areskutan (Ar) in Scandinavian Caledonides, and Pohorie (Po) in the Eastern Alps; ultra-high-T Kokchetav (Ko) Massif in Kazakhstan, Erzgebirge (Eg) in Germany, and Bohemian (Boh) rocks from this study. Grey ellipses show the error bars of the peak P–T estimates (see references for the peak conditions in Table 2). (**b**) P–T paths of ultra-high-T diamond-bearing UHP rocks and diamond (full symbols) crystallisation mechanism. Red field: possible melting region. Kokchetav PT path^28^ implies entrapment of fluid/melt inclusions by host minerals and diamond crystallization upon the retrograde decompression and cooling: subsequent phengite-out melting in graphite stability field causes diamond graphitization in the melt. Erzgebirge PT path^29^ suggests minor melt production on the prograde path due to phengite breakdown with insignificant diamond crystallization; more melting on retrograde path with entrapment of melt inclusions which crystallize diamond due to the decrease in carbon solubility upon decompression; no diamond graphitization on the retrograde path. Bohemian PT path^21^ suggests phengite breakdown and melting on the prograde path resulting in co-crystallization of diamond and peritectic phases; retrograde evolution without diamond graphitization.

Figure 6 examines nucleation media and triggers of microdiamond growth in UHP terranes worldwide for different peak conditions and P–T paths. The low-T rocks (~ 600 °C) with peak conditions below the wet solidus contain cuboidal diamonds inside aqueous fluid inclusions^6^. Diamond nucleation likely occurred on the prograde path upon crossing the graphite—diamond phase boundary (Fig. 6a) due to the lower solubility of diamond than graphite in fluid with the same carbon content^46^. Carbonates, readily soluble in aqueous fluids under high P, are the most likely source of carbon^6^.

The peak temperature estimates of 750–850 °C for HT diamond-bearing gneisses, which represent the most common UHP rocks, notably cluster just above the “wet” solidus for pelites below the 2CP (Fig. 6a). We propose that the transition from subsolidus fluid into hydrous silicate melt, accompanied by a decrease in carbon solubility, represents the most likely activation for diamond nucleation in these rocks (Fig. 6a). The cuboidal diamonds are associated with both fluid (CO_2_, CH_4_) and solid phases (carbonates, moissanite)^7,10,11^.

Nucleation and features of diamonds in UHT terranes are constrained by two phase boundaries, graphite–diamond and phengite-out melting curve, and the relative timing of their crossing on the prograde path (Fig. 6). The peak T increases from Kokchetav gneisses to Erzgebirge gneisses and further to the Bohemian rocks in this study. The prograde P–T path of Kokchetav gneisses (peak T ~ 1000 °C) enters the diamond stability field below the phengite-out reaction; it only crosses this reaction on the retrograde path^36^, outside of diamond stability (Fig. 6b). At the peak T, phengite is only partially consumed, as documented by preserved phengite inclusions in garnet^28^. At the same time, water content in the melt is relatively high at these conditions. CO_2_–H_2_O fluid inclusions within^48^ and associated with^14^ diamond either reflect the presence of fluids along the prograde path and close to the peak P–T conditions^14^, or represent a residuum after diamond crystallization from the melt with relatively high water content, as aqueous fluids cannot coexist with melts at supercritical conditions^37^. Diamonds occurring within multiphase solid inclusions (MSI), as well as intergranular diamonds^13^, crystallized from hydrous silicate-carbonate melt upon pressure decrease due to the drop in carbon solubility^49^. The contribution of metasomatism, fluid infiltration and melting, documented by field and isotopic evidence^50^, resulted in the complex morphology and variable carbon isotope record of diamonds in Kokchetav UHP rocks.

The temperature above 1000 °C of the Erzgebirge gneisses only slightly exceeds the phengite-out reaction and the melting of carbonated pelites in the diamond stability field, thus producing a minor amount of melt at peak P–T conditions. Therefore, diamond-bearing MSI^17^ represent silicate-carbonate melt produced by incongruent phengite melting and trapped as inclusions in peritectic phases on the prograde path. Residual silicate melt is preserved in inclusions in diamond^51^. Diamond could have crystallized inside these MSI along the retrograde path due to carbon solubility decrease. Light carbon isotopic values^52^ in the host metasediments reflect crustal (organogenic) carbon source.

In the Bohemian rocks from this study with peak temperatures > 1100 °C, above phengite breakdown, hydrous melt was produced in the diamond stability field, which triggered diamond co-crystallization with the peritectic phases (kyanite, garnet and zircon) close to peak P–T conditions (Fig. 6b). We demonstrate here that, exclusively, the Bohemian UHP-UHT terrain experienced sufficiently high T for melt formation along a prograde path and diamond nucleation at peak conditions, which are approximately 50 °C above the experimentally determined phengite breakdown at 4.5 GPa in pelitic systems. This confirms that the amount of melting greatly affects the efficiency of graphite to diamond conversion as shown by experiments^46^.

The peak P–T conditions for the exposed diamond-bearing HT-UHT terranes plot broadly along a subduction geotherm^20,36^, but cluster at melt-producing phase transitions (Fig. 6a). This provides evidence for the important role of melting not only in diamond nucleation but also in the exhumation of the terranes from mantle depths due to changes in rheological properties of the rocks. The predominance of high-T diamond-bearing gneiss occurrences is due to the lower and thus more easily reachable subduction depths required for diamond formation at lower T. At the same time, their exhumation is facilitated by a high amount of melt produced by water-saturated melting at the peak P–T conditions and during their exhumation along ITD path given the proximity of the wet solidus^53^. By contrast, the much rarer UHP-UHT diamond occurrences require a deeper subduction, and extreme temperatures for extensive water-absent melting through phengite breakdown at the peak, reached in the Bohemian Massif. At lower T, and incomplete phengite consumption^28^, decompressional melting^36^ facilitates the exhumation (e.g. Kokchetav Massif).

The presence of surface graphitization on microdiamonds from UHP terranes can serve as an indication of melting during the retrograde P–T path and exhumation in the graphite stability field, as graphitization develops when diamonds react with a melt but not with fluid^54^. Surface graphitization is a common feature of microdiamonds from Kokchetav UHP rocks^2^, which contain evidence for decompressional melting in the graphite stability field. Microdiamonds from the Bohemian rocks show no surface graphitization but only fractures filled with secondary graphite confirming no retrograde melting but presence of a late C–O–H fluid.

### Crystal morphology of microdiamonds as a proxy for subduction peak temperatures

Diamond morphology can provide further constrains on the conditions of diamond growth in different UHP rocks. Theory predicts that crystal morphology depends on two factors: the driving force determined by the degree of supersaturation and the growth kinetics determined by the efficiency of mass and heat transfer through the solvent^55^. Low driving force and/or fast carbon transfer promote an equilibrium growth of single octahedral diamonds with smooth crystal faces, whereas high driving force and/or slower carbon transfer result in growth of cuboid or cubo-octahedral diamonds with rough faces^55^. Temperature increase enhances carbon solubility in melts lowering the degree of supersaturation, and boosts the diffusion rate, which altogether promotes equilibrium growth.

Experiments demonstrate that higher temperature and the presence of water increases the diamond growth rate in carbonate and silicate melts by enhancing carbon transfer^46^. In “dry” melts the graphite to diamond transformation is relatively slow, often resulting in the formation of many crystallization centres that form aggregated low-quality crystals. In hydrous melts, a more efficient process controlled by a thermal gradient deposition produces monocrystalline diamonds of a larger size^56^, also due to a much lower nucleation rate. In addition, adsorption of H_2_O as an impurity during growth was proposed to inhibit growth of the {100} faces with increasing T^57^. Experiments conducted in H_2_O-bearing melts at T, greatly exceeding that of UHP-UHT rocks (above 1300–1400 °C), produced only octahedral diamonds^30,31,46^. By contrast, lower temperatures^31^ and/or “dry” melts^46^ shift the process towards formation of aggregated and even fibrous diamonds due to the decrease in the carbon transfer. The temperature effect on diamond morphology was examined in experiments with Ni–Fe–C melts^58^, which show a change from a lower-T cuboid to a higher-T octahedral crystal shape over a small temperature range of only 70–100 °C^57,59^. This explains the predominance of cubo-octahedra and scarcity of diamond octahedra in UHP-UHT terranes worldwide (See Table 1) in contrast to predominant octahedra (or their rounded secondary forms e.g. dodecahedra or tetrahexahedra) and subordinate cubo-octahedra in cratonic diamonds sampled by kimberlites^60,61^, which formed at more extreme P and T than UHP rocks^62^.

Octahedral morphology of microdiamonds in rock A implies an equilibrium growth at a low degree of supersaturation, and the hydrous silicate-carbonate melt which formed due to decomposition of phengite is a suitable medium for such growth. By contrast, the cubo-octahedral (± polycrystalline) diamonds in the rock B indicate a higher nucleation rate. While this may be due to lower temperature, the precision of the peak P–T estimates for the two rock types is insufficient to evaluate the effect of temperature on diamond morphology. Apart from temperature, phase transitions can also increase the degree of supersaturation. The P–T peak estimate for the rocks in this study is located close to the miscibility gap between carbonate and silicate-carbonate melts^44,45^, offering another possible explanation for the observed change in diamond morphology towards more irregular diamond forms due to different carbon solubility in silicate and carbonate melts^56^.

## Conclusions

We present a new model for the formation of microdiamonds in UHP metamorphic rocks, which explains a variety of worldwide occurrences. It is based on a combined study of uniquely preserved Bohemian microdiamonds (morphology, resorption and carbon isotope composition) and their host rocks, including peak P–T estimates.

1. We demonstrate that diamond nucleation and growth is triggered by phase transitions related to changes in water activity of the host rocks including crossing the wet solidus of pelites, phengite-out melting, and silicate-carbonate immiscibility, where hydrous melt provides the medium for diamond nucleation and growth along the prograde metamorphic path.
2. The clustering of the peak P–T estimates for the diamond-bearing UHP terranes along the melt-producing phase transitions provide evidence for the important role of partial melting, not only in diamond nucleation but also during initial exhumation of deeply buried rocks. Numerous occurrences of diamond-bearing gneisses which experienced temperatures of 750–850 °C can be explained by a higher-volume water-present melting, thus facilitating their exhumation. In addition, formation of these rocks does not require any extreme subduction depth and heat flow, in contrast to the UHP-UHT ones.
3. We established for the first time a relationship between the temperature of UHP rocks and the crystal morphology of microdiamonds. Most UHP terrains contain cubic, cubo-octahedral, and polycrystalline diamonds. Smooth-faced octahedra are extremely rare in UHP rocks and suggest extreme peak temperature (> 1100 °C), which are common in the case of cratonic diamonds.
4. The temperature dependence of diamond crystal morphology provides a method for better constraining the close-to-peak P–T evolution of UHP rocks and deep subduction and exhumation processes. Our results might also explain the formation of different morphological populations of kimberlitic diamonds, where cubo-octahedral diamonds may represent products of lower-T metasomatism compared to higher-T metasomatism producing octahedral diamonds.

## Methods

### Sample preparation

Thin sections for optical microscopy and SEM imaging of diamonds were polished using Al_2_O_3_ abrasives to avoid contamination by synthetic diamond from polishing material. Final hand-polishing using OP-U silica suspension of 0.04 µm grain size (Struers) was carried out so that the diamonds stand out above the surface of host minerals. No diamond polishing was applied.

### Scanning electron microscopy

Samples coated with gold were imaged using SEM MIRA3 equipped with BSE, both in SE and BSE modes at TESCAN in Brno. Accelerating voltage ranged between 15 and 25 kV.

### AFM

Atomic Force Microscopy (AFM) measurements were performed in the Czech Metrology Institute using Dimension Icon microscope (Bruker) in ScanAsyst mode with Scanasyst-Air probes. AFM data were processed using software Gwyddion^63^.

### FIB-TEM

Electron transparent foils were prepared for transmission electron microscopy (TEM) applying the site-specific focused-ion-beam (FIB) technique that allows cutting an electron-transparent foil from pre-selected areas of interest. The TEM foils were 15–20 μm wide, 10–15 μm deep and approximately 150 nm thick. Details of the technique are given in^64,65^. Analytical and high-resolution transmission electron microscopy (ATEM, HRTEM) using a FEI Tecnai™ G2 F20 X-Twin at GeoForschungs Zentrum (GFZ) in Potsdam, operated at 200 kV with a field emission gun (FEG) electron source, was used for the present study. The TEM is equipped with a Gatan imaging filter (GIF Tridiem) allowing energy-filtered imaging. ATEM was performed with an EDAX X-ray analyser equipped with an ultra-thin window. The X-ray intensities were measured in scanning transmission mode (STEM) where the electron beam was scanned over a pre-selected area, minimizing mass loss during data acquisition.

### Carbon isotope analyses

Carbon isotope compositions of diamond inclusions were analysed by secondary ion mass spectrometry (SIMS) using a CAMECA IMS1280 instrument (Nordsim facility, Stockholm). A ca. 1.3 nA, 20 keV impact energy Cs^+^ primary beam was focussed in Gaussian mode and rastered during analysis over a 5 × 5 μm area, resulting in a spot size of ca. 10 μm. Potential sample charging effects due to the small size of the inclusions embedded in a non-conducting matrix were minimised using a low energy normal incidence electron flooding gun. Secondary C^+^ ions were centred in the 2000 μm field aperture (field of view on the sample of ca. 20 μm) automatically at the start of each analysis, passed through a 50 eV energy window and separated using a magnetic field that was locked to high stability using an NMR field sensor. Simultaneous measurement of ^12^C and ^13^C signals was performed over 16 cycles of 4 s integrations in low-noise Faraday detectors. In order to eliminate interference of the ^12^C^1^H^+^ species on ^13^C^+^, the axial Faraday detector was operated at a nominal mass resolution (M/ΔM) of 4000, with the off-axis detector measuring ^12^C^+^ at M/ ΔM of 2500. Data are reported as δ ^13^C_PDB_ values in parts per thousand (‰), where PDB refers to the carbon isotope composition of the Pee Dee belemnite; for measured carbon isotope ratios, δ^13^C_PDB_ = δ ^13^C_std_ + δ ^13^C_PDB_(std) + 10^–3^ × δ ^13^C_std_ x δ ^13^C_PDB(std)_. An in-house pyrolised graphite disk (C-pyr2) with a δ ^13^C value of -35.70 ‰ was used to correct for instrumental mass bias. This same reference material has previously been reported as yielding an indistinguishable δ ^13^C value^66^ (their Table S1) when calibrated against a synthetic diamond (SYNAL, with δ ^13^C = − 23.9‰^67^), demonstrating that a graphite reference material of this type can be used with confidence to determine δ ^13^C in diamond.

## Supplementary Information

Supplementary Information
