# Supplementary material for: Metamorphic microdiamond formation is controlled by water activity, phase transitions and temperature
Source: Sci Rep. 2021 Apr 8;11:7694. doi: 10.1038/s41598-021-87272-1 (PMC8032753; doi:10.1038/s41598-021-87272-1)
Supplement: Supplementary file 1 — Supplementary Information [file 41598_2021_87272_MOESM1_ESM.pdf]

Supplementary information

Microdiamond formation is controlled by water activity, phase transitions and temperature

J. Kotková[1,2*], Y. Fedortchouk[3], R. Wirth[4], M. J. Whitehouse[5]

[1]Czech Geological Survey, Klárov 3, 118 21 Prague 1, Czech Republic, jana.kotkova@geology.cz

[2]Department of Geological Sciences, Masaryk University, Kotlářská 2, 611 37 Brno, Czech Republic

[3] Department of Earth and Environmental Sciences, Dalhousie University, Halifax, Canada, B3H 4R2

[4] Interface Geochemistry, GFZ German Research Centre For Geosciences, Telegrafenberg, C-120, 14473, Potsdam, Germany

[5]Department of Geosciences, Swedish Museum of Natural History, Box 50007, SE-104 05 Stockholm, Sweden

*corresponding author

Contents

Supplementary Table S1: SIMS carbon isotope data from diamond inclusions and standards.

Supplementary Figure S1. Additional SE and BSE images of diamonds selected for FIB-TEM analysis.

Supplementary Table S2: Summary of FIB-TEM foils studied and TEM data acquired.

Table S1 SIMS carbon isotope data from diamond inclusions and standards

Analysis ID	Seq.[a]	$^{13}C/^{12}C^{b}$	σ_{intl} (%)	^{12}C intensity (cps x 10^6)	^{12}C cps/ av. std	$\delta^{13}C_{PDB} \pm 2\sigma_{extl}$ (‰)
Diamond inclusions						
n4979_c-G1-A11	7	0.010586	0.007	457	0.95	-29.4 ± 0.4
n4979_c-G1-C2	8	0.010581	0.017	344	0.72	-29.9 ± 0.4
n4979_c-G1-C9	9	0.010541	0.018	164	0.34	-33.5 ± 0.6
n4979_c-G1-C9a	10	0.010578	0.018	275	0.57	-30.2 ± 0.6
n4979_c-G1-D4	11	0.010616	0.015	342	0.71	-26.7 ± 0.4
n4979_c-G1-E1	13	0.010612	0.017	296	0.62	-27.0 ± 0.6
n4979_c-G2-A11	14	0.010556	0.028	233	0.49	-32.2 ± 0.6
n4979_c-G2-D8	15	0.010562	0.013	330	0.69	-31.6 ± 0.4
n4979_c-G3-A3	16	0.010608	0.008	409	0.85	-27.4 ± 0.4
n4979_c-G4-B1	17	0.010621	0.016	344	0.72	-26.2 ± 0.4
n4980_c-H5	22	0.010666	0.016	318	0.65	-22.0 ± 0.4
n4980_c-I8	23	0.010677	0.013	511	1.05	-21.1 ± 0.4
Reference material analyses[c]						
Cpyr-y2_mt1301_@1	1	0.010517	0.008	492		-35.7 ± 0.4
Cpyr-y2_mt1301_@2	2	0.010520	0.006	498		-35.5 ± 0.4
Cpyr-y2_mt1301_@3	3	0.010518	0.010	520		-35.6 ± 0.4
Cpyr-y2_mt1301_@4	4	0.010516	0.013	492		-35.8 ± 0.4
Cpyr-y2_mt1301_@5	5	0.010517	0.010	490		-35.7 ± 0.4
Cpyr-y2_mt1301_@6	6	0.010517	0.010	480		-35.8 ± 0.4
Cpyr-y2_mt1301_@7	18	0.010516	0.010	522		-35.9 ± 0.4
Cpyr-y2_mt1301_@8	19	0.010514	0.014	492		-36.0 ± 0.4
Cpyr-y2_mt1300_@1	20	0.010519	0.007	476		-35.6 ± 0.4
Cpyr-y2_mt1300_@2	21	0.010520	0.010	486		-35.4 ± 0.4

Notes: [a]Run sequence number. [b]Drift corrected, measured $^{13}C/^{12}C$ ratio, uncorrected for instrumental mass bias. [c]Reference material C-pyr2, $\delta^{13}C_{PDB}$ = -35.4 **‰.**

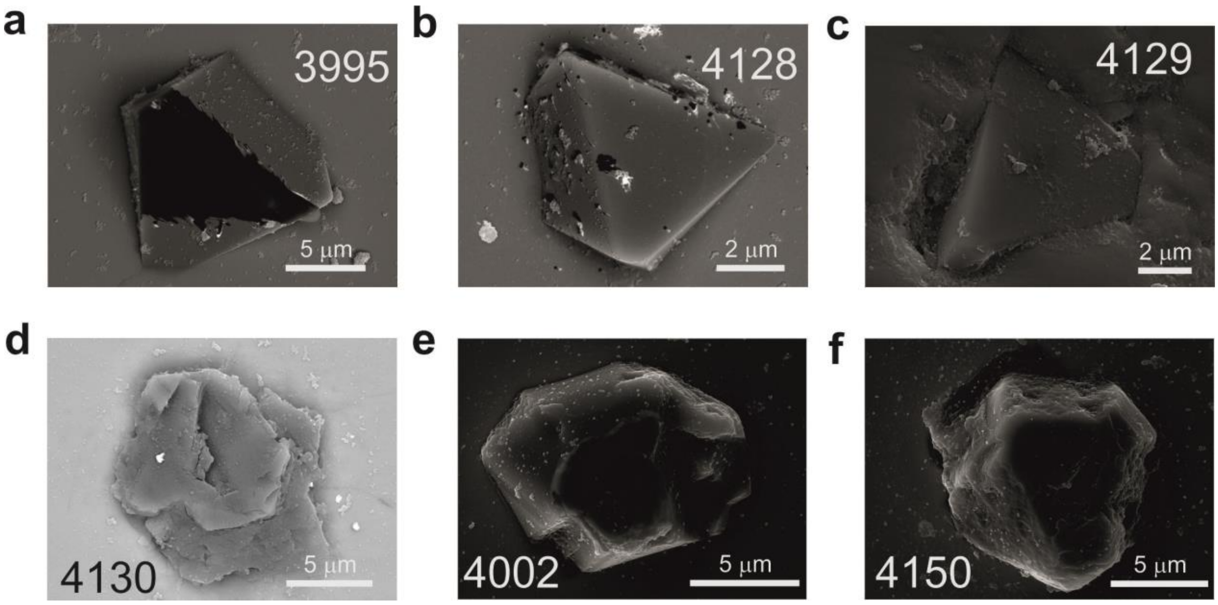

Figure S1. Additional SE and BSE images of diamonds selected for FIB-TEM analysis. Octahedral diamonds enclosed in kyanite (rock A; a-c), diamond cuboid enclosed in garnet (d) and zircon (e,f) in the rock B. Four-digit numbers refer to FIB-TEM sample numbers. Dust captured on the faces due to imperfect Au coating.

Table S2. Summary of FIB-TEM foils studied and TEM data acquired

foil No	morphology	host	other phases	analysed melt	composition
rock A					
3995	octahedron	Ky	Qtz, Wm, Mg-ferrite	interface	Ca, Mg, Fe, Cl, S
3997	octahedron	Ky			
4128	octahedron	Ky	Gr		
4129	octahedron	Ky		void at interface	
rock B					
4002	cuboid	Zrn		interface, triangular gap	Ca, K, S
				interface, elong gap	Ca, K, Al, Cl
4010	cuboid	Grt	Chl		
4012	cuboid	Grt	Gr	interface, triangular gap	Ca, Fe, S
					Ca, Cl
4130	cuboid, polycryst.	Grt	Rt, Qtz		
4150	cuboid, polycryst.	Zrn	Gr	interstitial	Zn, Fe, S
					Cl, K, Zn
					Na, Cl, Zn, S

Ky - kyanite, Grt - garnet, Zrn - zircon; Wm - white mica, Qtz - quartz, Gr - graphite, Chl - chlorite, Rt - rutile.
